# Oral Microbiome Alterations Associated with Early Childhood Caries Highlight the Importance of Carbohydrate Metabolic Activities

**DOI:** 10.1128/mSystems.00450-19

**Published:** 2019-11-05

**Authors:** Yuan Wang, Sa Wang, Chunyan Wu, Xi Chen, Zhuhui Duan, Qian Xu, Wen Jiang, Lei Xu, Tingting Wang, Lingkai Su, Ying Wang, Yadong Chen, Jie Zhang, Yun Huang, Suman Tong, Cheng Zhou, Shuli Deng, Nan Qin

**Affiliations:** a Department of Conservative Dentistry and Periodontics, Affiliated Hospital of Stomatology, Zhejiang University School of Medicine, Hangzhou, China; b Shanghai Tenth People’s Hospital Affiliated to Tongji University, Shanghai, China; c Realbio Genomics Institute, Shanghai, China; d State Key Laboratory for Diagnosis and Treatment of Infectious Disease, The First Affiliated Hospital, Zhejiang University School of Medicine, Hangzhou, China; University of Naples Federico II

**Keywords:** early childhood caries, metagenomics, oral microbiome, functional profile, preschool children

## Abstract

Globally, dental caries is the most prevalent chronic oral disease and affects roughly half of all children. The aim of this report was to use metagenomic analyses to investigate the relationship between the oral microbiome and caries in preschool children. A total of 25 preschoolers, aged 3 to 5 years old with severe early childhood caries (ECC), and 19 age-matched, caries-free children as controls were recruited. Saliva samples were collected from the participants and were subjected to metagenomic analyses, whereby the oral microbial communities were investigated. The metagenomic analyses revealed substantial microbiota differences between the two groups, indicating apparent shifts of the oral microbiome present in the ECC group. At the species level, the ECC-enriched microbes included Prevotella amnii, Shuttleworthia satelles, Olsenella uli, and Anaeroglobus geminatus. Interestingly, Actinomyces odontolyticus and Actinomyces graevenitzii exhibited apparent differences at the strain level but not the species level between the ECC and control groups. Functional examination showed that the ECC group displayed extensive alterations in metabolic genes/pathways/modules, including enriched functions in sugar metabolism. Finally, an SVM (support vector machine) classifier comprising seven species was developed and generated a moderately good performance in predicting caries onset (area under the receiver operating characteristic curve [AUC] = 78.33%). Together, these findings indicate that caries is associated with considerable changes in the oral microbiome, some of which can potentially be exploited as therapeutic targets or diagnostic markers. (This study has been registered at ClinicalTrials.gov under registration no. NCT02341352.)

**IMPORTANCE** Dental caries is a highly prevalent oral disease that can lead to severe dental damage and may greatly compromise the quality of life of the affected individuals. Previous studies, including those based on 16S rRNA gene, have revealed that the oral microbiota plays a prominent role in development of the disease. But the approach of those studies was limited in analyzing several key microbiome traits, including species- or strain-level composition and functional profile. Here, we performed metagenomic analyses for a cohort of preschool children with or without caries. Our results showed that caries was associated with extensive microbiota differences at various taxonomic and functional levels. Some caries-associated species had not been previously reported, some of which may have significant clinical implications. A microbiome gene catalogue from children with caries was constructed for the first time. The results demonstrated that caries is associated with alterations of the oral microbiome, including changes in microbial composition and metabolic functional profile.

## Abstract (precis)

Dental caries is a highly prevalent oral disease that can lead to severe dental damage and may greatly compromise the quality of life of the affected individuals. Previous studies, including those based on 16S rRNA gene, have revealed that the oral microbiota plays a prominent role in development of the disease. But the approach of those studies was limited in analyzing several key microbiome traits, including species- or strain-level composition and functional profile. Here, we performed metagenomic analyses for a cohort of preschool children with or without caries. Our results showed that caries was associated with extensive microbiota differences at various taxonomic and functional levels. Some caries-associated species had not been previously reported, some of which may have significant clinical implications. A microbiome gene catalogue from children with caries was constructed for the first time. The results demonstrated that caries is associated with alterations of the oral microbiome, including changes in microbial composition and metabolic functional profile.

## INTRODUCTION

Dental caries, also known as tooth decay, is one of the most common oral infections in children (1). It is a destructive process that causes decalcification of tooth enamel and subsequently leads to continued breakdown of enamel and dentin (2). If left untreated, some pathogens or pathobionts in the oral microbiota can penetrate the enamel and dentin to reach the pulp, which leads to pulpitis and periapical periodontitis. In the absence of immediate and effective infection control, these local infections may expand and progress to culminate in more serious conditions, such as cellulitis (3, 4), osteomyelitis (5), bacteremia, and bacterial endocarditis (6).

The Global Burden of Disease (GBD) study reported that caries affected more than 10% of the world’s population in 2015 and that the incidence of deciduous caries increased by 5.6% between 2005 and 2015 (7). According to an oral epidemiological investigation in China in 2018, there is a marked increase in the prevalence of childhood caries, up from 5.8% a decade ago (8). Therefore, study of the pathogenesis of childhood caries is of great significance in prevention, screening, and early intervention for vulnerable or affected children.

In previous studies, we performed 16S rRNA gene amplicon sequencing to examine the bacterial microbiota of dental plaques to study the microbial traits in severe cases of early childhood caries (ECC) (9), which revealed dynamic changes of oral microbiota at different stages of caries progression (10, 11). Nevertheless, this approach cannot provide some key information about oral microbiota, such as species-level and strain-level resolution and metabolic profile, which are likely important for caries pathogenesis (12).

Fang Yang et al. employed a microbial functional gene microarray to reconstruct the functional profiles of human saliva microbiota for healthy and caries-active adults; the results showed that saliva microbiota carried disease-associated functional signatures, which could be potentially exploited as diagnostic markers (13). However, the functional features of gene microarrays are dependent on preselected probe sets, thus limiting their scope in functional dissection of microbial communities.

In this study, we analyzed the oral microbiome in preschoolers, whereby a gene catalogue was constructed for children with ECC. Our results not only corroborated previous findings that the microbiome has a great relevance in the occurrence of dental caries but also revealed new microbial species and functional groups associated with the disease.

## RESULTS

### Sample collection, sequencing, and quality control.

Saliva samples were collected from 25 preschool children with severe early childhood caries (ECC) (decayed, missing, and filled tooth surfaces [dmfs] ≥ 8) and 19 healthy control subjects (dmfs = 0) living in Lin’an, Zhejiang Province (see Table S1 in the supplemental material). There were no differences in age, gender, or body mass index (BMI) between the caries group and the healthy group. A total of 195-GB of raw data was generated from the Illumina HiSeq 2000 platform. After filtering out low-quality data and host contamination, an average of 3.08 GB (1.51 to 7.07 GB) of clean data were generated for each sample (Table S2).

10.1128/mSystems.00450-19.3 TABLE S1 Phenotype information of the Chinese children in our research (44 samples). Download Table S1, XLSX file, 0.02 MB. Copyright © 2019 Wang et al. 2019 Wang et al. This content is distributed under the terms of the Creative Commons Attribution 4.0 International license.

10.1128/mSystems.00450-19.4 TABLE S2 Data production of 44 samples in our research. Download Table S2, XLSX file, 0.01 MB. Copyright © 2019 Wang et al. 2019 Wang et al. This content is distributed under the terms of the Creative Commons Attribution 4.0 International license.

To examine the association between oral microbiota and ECC development, we classified the 19 healthy children into two subgroups based on the changes in caries state during the 6 months after the initial sampling: (i) the “stay healthy” (H2H) subgroup, in which the 15 subjects maintained a healthy state, and (ii) the “caries-onset” (H2C) subgroup, in which the 4 subjects underwent the transition from a healthy to a caries-active state.

### Shifts of the oral microbiomes in preschoolers with caries.

After filtering out 27.8% ± 16.7% sequences/reads as host gene sequences, approximately 49.8% ± 3.8% of the reads from each sample were mapped to 7,312 reference bacterial genomes from GenBank and the HMP (Human Microbiome Project).

To investigate the diversity of salivary microbiome richness, the Shannon-Weiner index and Simpson index were calculated for species and genes, which showed that microbial diversity and richness were similar between the ECC and control groups (*P* > 0.05) (Fig. S1a). To assess microbial structure alterations in the ECC group, we employed nonparametric analyses and principal-coordinate analysis (PCoA). Three nonparametric methods were applied, namely, the multiresponse permutation procedure (MRPP), analysis of similarity (ANOSIM), and permutational multivariate analysis of variance (Adonis). Apparent differences were detected by both the MRPP and Adonis at the phylum, class, order, family, and genus levels (*P < *0.05) or by the ANOSIM at the phylum, family, and genus levels (*P < *0.05). PCoA based on the Bray-Curtis distance of species abundance showed that the ECC and healthy groups displayed apparent microbiome structural differences (Fig. S1c).

10.1128/mSystems.00450-19.1 FIG S1 Diversity comparisons of the oral microbiome between the ECC group and the healthy group. (a) Box plot of richness, Shannon index, and Simpson index of species and genes are shown. (b) Venn diagram comparing the oral microbiome gene sets between patients with dental caries and healthy controls. (c) PCoA plot with Bray-Curtis distances generated from species abundance is shown. Download FIG S1, PDF file, 0.8 MB. Copyright © 2019 Wang et al. 2019 Wang et al. This content is distributed under the terms of the Creative Commons Attribution 4.0 International license.

**FIG 1:**
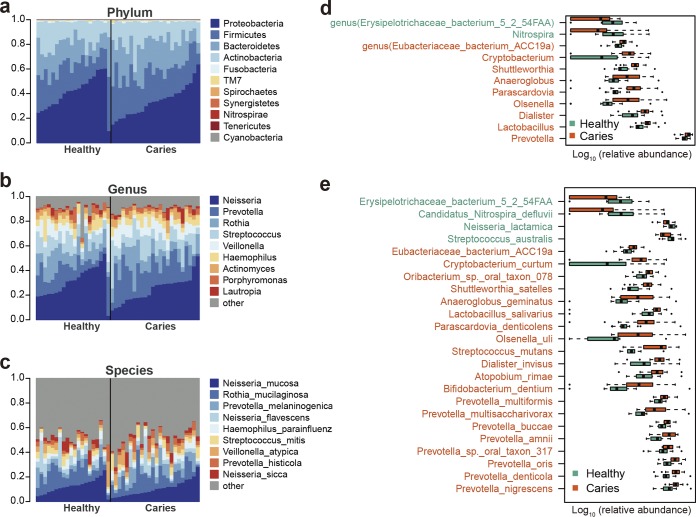
Relative abundances of phylotypes in healthy and ECC (caries) groups. (a to c) Relative abundances of phyla, genera, and species, respectively, are shown in a bar plot. (d and e) Relative abundances of genera (d) and species (e) with significantly different abundances (FDR < 0.1) are shown in a box plot.

At the phylum level, *Proteobacteria*, *Firmicutes*, *Bacteroidetes*, and *Actinobacteria* were the most abundant taxa in both groups (Fig. 1a). The healthy group displayed a higher abundance of *Nitrospirae* than did the ECC group (Wilcoxon test, false discovery rate [FDR] < 0.1) (Table S3).

10.1128/mSystems.00450-19.5 TABLE S3 Detailed information of caries/healthy group-associated phylum, genus, and species (FDR < 0.1). Download Table S3, XLSX file, 0.04 MB. Copyright © 2019 Wang et al. 2019 Wang et al. This content is distributed under the terms of the Creative Commons Attribution 4.0 International license.

At the genus level, *Neisseria*, *Prevotella*, *Rothia*, *Streptococcus*, *Veillonella*, and *Haemophilus* were among the major phylotypes in both groups (Fig. 1b). Subsequent analysis of relative abundance revealed that nine genera, including *Prevotella*, were more abundant in the ECC group than in the healthy group, whereas *Nitrospira* and the genus *Erysipelotrichaceae bacterium* 5_2_54FAA were enriched in the healthy group (Fig. 1d; Table S3).

At the species level, Neisseria mucosa, Rothia mucilaginosa, and Prevotella melaninogenica accounted for large shares of the total microbial abundance in both the children with caries and caries-free children, suggesting that these microbes belonged to the stable oral microflora (Fig. 1c). The caries-free subjects exhibited an increased relative abundance of Neisseria lactamica or Streptococcus australis (Fig. 1e; Table S3). Conversely, 20 species were found to be enriched in the severe ECC group (Fig. 1e; Table S3). These species included Streptococcus mutans (14–17) and multiple *Prevotella* spp. (12), which have been reported to be associated with dental caries, as well as Prevotella amnii, Shuttleworthia satelles, Olsenella uli, and Anaeroglobus geminatus, whose connections to the disease have not been reported.

To further delineate features of the ECC-associated saliva microbiome, we identified 26,264 differentially abundant genes (Wilcoxon rank sum test, FDR < 0.07) and clustered them into metagenomic species (MGS) on the basis of their correlated abundance variation across samples. We grouped the differentially abundant genes into 18 MGS, with 12 MGS enriched in the ECC group and 6 MGS enriched in the healthy controls (Fig. S2). Of the 12 MGS enriched in the ECC group, four were *Prevotella* species. On the other hand, two *Neisseria* species were more abundant in healthy subjects (Fig. S2). Importantly, these MGS-based results were in agreement with those derived from taxonomic analysis (Fig. 1d and e).

10.1128/mSystems.00450-19.2 FIG S2 Differentially abundant MGS in caries (*n* = 12) and healthy individuals (*n* = 6). A heat map of 25 “tracer” gene abundances for each MGS in the caries group (25 individuals) and the healthy group (19 individuals) is shown. Individuals are shown in columns, sorted by increasing abundance of the caries-enriched MGS. The abundance of genes in rows is indicated by a color gradient (white, not detected), and the enrichment significance is shown with a *P* value. Download FIG S2, PDF file, 0.8 MB. Copyright © 2019 Wang et al. 2019 Wang et al. This content is distributed under the terms of the Creative Commons Attribution 4.0 International license.

### Strain-level variations of the caries and healthy subjects.

**FIG 2:**
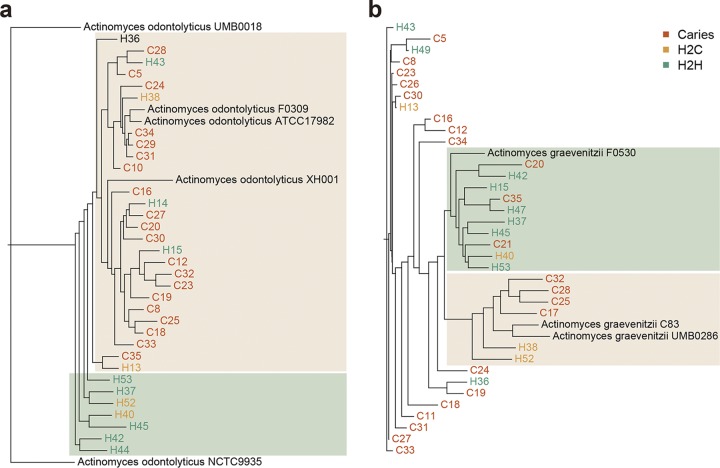
Strain-level phylogenetic trees of *A. odontolyticus* (a) and *A. graevenitzii* (b) of ECC (caries) and healthy (H2H and H2C) group samples. Available reference genomes were included in the phylogenetic trees.

It is being increasingly recognized that microbial species consist of distinct strains and that strain-level variations are a crucial factor for determining the functions of microbial communities. To examine the strain-level variants between the caries and healthy groups, we applied StrainPhlAn, an assembly-free strain-level phylogenetic method that identifies single nucleotide variants (SNVs) in species-specific marker genes (18). Using the SNV-based analysis, we built the phylogenetic trees of the common species from the samples with sufficient coverage and available reference genomes. We found considerable strain-level heterogeneity between the caries and healthy groups in two species, i.e., Actinomyces odontolyticus and Actinomyces graevenitzii, albeit neither of which displayed a difference in relative abundance at the species level. For *A. odontolyticus* (Fig. 2a), the dominant strains in caries individuals were phylogenetically closer to *A. odontolyticus* F0309, *A. odontolyticus* ATCC 17982, and *A. odontolyticus* XH001 than those in the control group. In addition, the dominant *A. graevenitzii* strains in the ECC and H2C subjects were closely related to *A. graevenitzii* C83 and *A. graevenitzii* UMB0286 strains, as were those in the healthy subjects to *A. graevenitzii* F0530 (Fig. 2b).

### Cooccurrence networks of saliva microbiota under healthy and ECC conditions.

**FIG 3:**
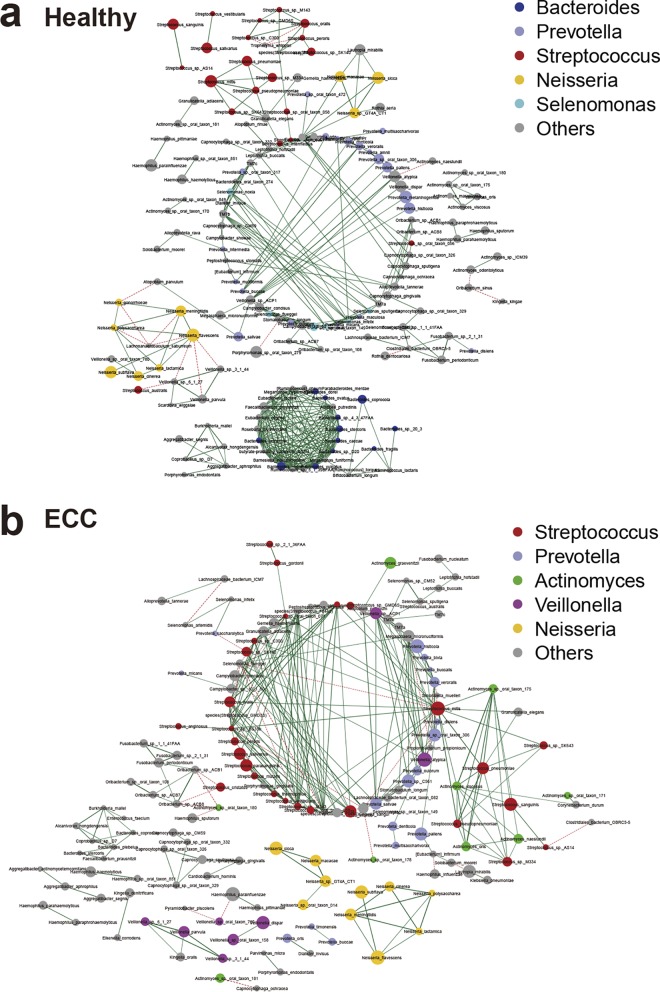
Networks in oral microbial communities under ECC and healthy conditions are shown, with each microbial species and cooccurrence relationship indicated by a node and an edge, respectively. A connection (line between dots) indicates a strong (Spearman’s ρ > 0.6) and significant (FDR < 0.05) correlation. The size of each node is proportional to the relative abundance. Lines between nodes indicate positive correlations (green) or negative correlations (red). The top five abundant genera are indicated in color.

To analyze the patterns of interbacterial interactions in oral microbial communities under healthy and ECC conditions, we constructed the cooccurrence networks for the two groups, respectively. We inferred the metacommunity cooccurrence networks based on Spearman correlation relationships and *P* values for correlations adjusted with the FDR (Benjamini and Hochberg). This generated a metacommunity cooccurrence network of the ECC group comprising 282 edges, reflective of the interbacterial associations, among 150 species/strains (Fig. 3a), as well as a network of the healthy group containing 374 edges among 164 species/strains (Fig. 3b). Whereas the healthy and ECC groups shared a considerable proportion of the edges (17.65% for the healthy network and 24.40% for the ECC network), most edges were condition specific (82.35% for the healthy network and 76.60% for the ECC network). In other words, approximately 20% of the interbacterial associations were shared by the two groups.

We calculated topological features for each node in the networks with the igraph package. This feature set included betweenness centrality (the number of shortest paths going through a node), closeness centrality (the number of steps required to access all other nodes from a given node), and degree (the number of adjacent edges). Comparing these features between the two networks, we found that the closeness of nodes in the healthy group network was significantly higher than that in the ECC group network (*P < *4E−11, Wilcoxon rank sum test), whereas degree and betweenness were not significantly different between the two groups.

Notably, there were one and two main clusters with >10 nodes in the networks of ECC and control groups, respectively. *Bacteroides* spp. and *Prevotella* spp. were dominant in the two main clusters of the control group, as were *Streptococcus* spp. and *Prevotella* spp. in the main cluster of the ECC network.

### Functional profiles of caries and healthy subjects.

To construct the gene catalogue, we combined the genes predicted from the assembled contigs and genes from the Human Oral Microbiome Database (HOMD). After filtering redundant genes, we generated a nonredundant oral microbial gene catalogue containing 2,200,443 genes. The healthy and ECC groups shared 107,151 genes, representing 71.05% and 81.58% of their core genes, respectively (Fig. S1b).

To investigate the functional role of the oral microbiome in ECC, we annotated the oral gene catalogues using KEGG (Kyoto Encyclopedia of Genes and Genomes database) and eggNOG (evolutionary genealogy of genes: Nonsupervised Orthologous Groups database). Correspondingly, three types of functional profiles were generated and compared between the caries and control groups: (i) gene profile, (ii) KEGG orthology profile, and (iii) eggNOG profile.

**FIG 4:**
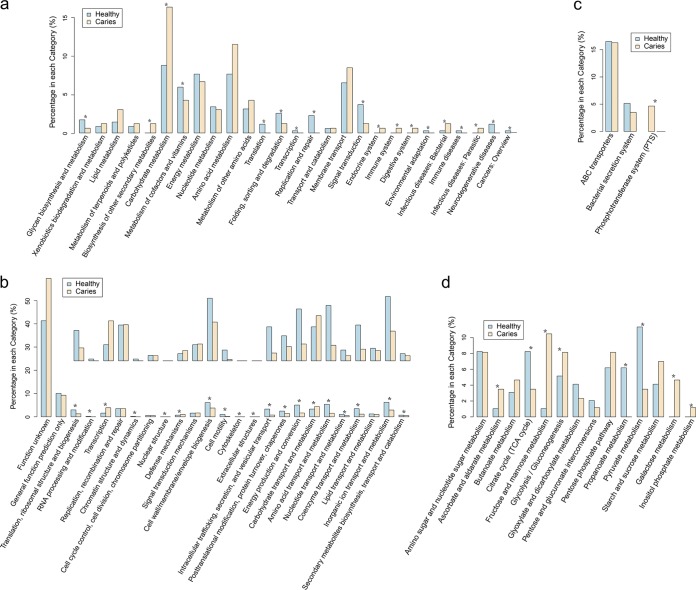
Functional distribution of KEGG orthologous genes and eggNOG orthologous genes enriched in healthy and ECC (caries) children. (a) Comparison between the KEGG orthologous genes enriched for healthy and ECC children for each KEGG functional category at the second functional level. (b) Comparison between the eggNOG orthologous genes enriched for healthy and ECC children for 24 eggNOG orthologue group functional categories. (c and d) Comparison between KEGG orthologous genes for healthy and ECC children for each KEGG functional category at the third functional level: membrane transport (c) and carbohydrate metabolism (d). Asterisks indicate hypergeometric distribution test results with phyper.R (*, FDR < 0.05).

Analysis of the gene profile revealed a skewed pattern such that 1,200 genes were enriched in the ECC group, as opposed to only 62 genes in the healthy group (Wilcoxon rank sum test, FDR < 0.07) (Table S4). Using a 0.85 identity threshold, these genes were mapped to GenBank via BLAT. Annotation of the differentially abundant genes to the KEGG and eggNOG databases revealed that extensive differences were present between the two groups in a variety of functions/pathways, including a relatively increased level of carbohydrate metabolism and decreased levels of translation, energy metabolism, coenzyme/cofactor/vitamin metabolism, and signal transduction in the caries group (Fig. 4a and b). Differences were also detected at the third-level components of KEGG between the two groups. In the membrane transport pathway, the phosphotransferase system was enriched in the ECC children (Fig. 4c). In the carbohydrate metabolism pathway, citrate cycle (tricarboxylic acid [TCA] cycle) was enriched in healthy children, while glycolysis/gluconeogenesis was enriched in the ECC group (Fig. 4d). Notably, the glucosyltransferase (GTF) gene (12_gene_id_1342) (Table S4) showed an increased relative abundance in ECC subjects, as did modules of the AI-2 (autoinducer-2) transport system, phosphotransferase (PTS) system, glucitol/sorbitol-specific II component, and nucleotide sugar biosynthesis (Table S5).

10.1128/mSystems.00450-19.6 TABLE S4 Detailed information of caries and healthy group-associated genes. Download Table S4, XLSX file, 0.8 MB. Copyright © 2019 Wang et al. 2019 Wang et al. This content is distributed under the terms of the Creative Commons Attribution 4.0 International license.

10.1128/mSystems.00450-19.7 TABLE S5 Detailed information of crucial caries and healthy group-associated modules. Download Table S5, XLSX file, 0.01 MB. Copyright © 2019 Wang et al. 2019 Wang et al. This content is distributed under the terms of the Creative Commons Attribution 4.0 International license.

### Host factors associated with some microbial taxa and pathways.

Permutational multivariate analysis of variance (PERMANOVA) was performed to analyze the association between clinical factors and interpersonal distance (Bray-Curtis) of microbial composition (Table S6). For the 44 children, 3 factors were associated with interpersonal distance of microbial composition (*P < *0.05). Education background, height, and caries status were the major sources of variance in the microbial species composition. In contrast, BMI, gender, income level, and dietary habit made comparatively minor or nonsignificant contributions to oral microbiome composition. We performed multivariate linear association analyses between the host clinical phenotypes and 205 representative species (>0.01% of total microbial reads and present in at least 10 individuals) or 315 MetaCyc pathways. When corrections were made for age and gender, we identified 28 associations with an FDR of <0.1 between 3 factors and 24 species (Table S7), as well as 52 associations between 5 factors and 176 MetaCyc pathways (Table S8). In our study, toothache in the past year was correlated not only with microbial composition but also with MetaCyc pathways. The relative abundances of Prevotella amnii, Prevotella buccae, and Streptococcus mutans were positively correlated with toothache in the past year and caries status. Other associations with phenotypical variables included a negative correlation between Neisseria lactamica and toothache in the past year and caries status. MetaCyc pathways, including biotin biosynthesis II, purine nucleobase degradation, and guanosine nucleotide degradation, were positively correlated with toothache in the past year and the frequency of dietary intake of biscuits, cakes, and bread. In comparison, the prevalence of l-lysine biosynthesis was inversely associated with decayed, missing, or filled tooth (dmft), dmfs, and toothache in the past year.

10.1128/mSystems.00450-19.8 TABLE S6 PERMANOVA for the influence of host clinical phenotype on the taxonomic and pathway profiles. Download Table S6, XLSX file, 0.01 MB. Copyright © 2019 Wang et al. 2019 Wang et al. This content is distributed under the terms of the Creative Commons Attribution 4.0 International license.

10.1128/mSystems.00450-19.9 TABLE S7 Significant associations with individual species (FDR < 0.1). Download Table S7, XLSX file, 0.01 MB. Copyright © 2019 Wang et al. 2019 Wang et al. This content is distributed under the terms of the Creative Commons Attribution 4.0 International license.

10.1128/mSystems.00450-19.10 TABLE S8 Significant associations with individual MetaCyc pathway (FDR < 0.1). Download Table S8, XLSX file, 0.01 MB. Copyright © 2019 Wang et al. 2019 Wang et al. This content is distributed under the terms of the Creative Commons Attribution 4.0 International license.

### Disease classification based on oral microbiota profiles.

**FIG 5:**
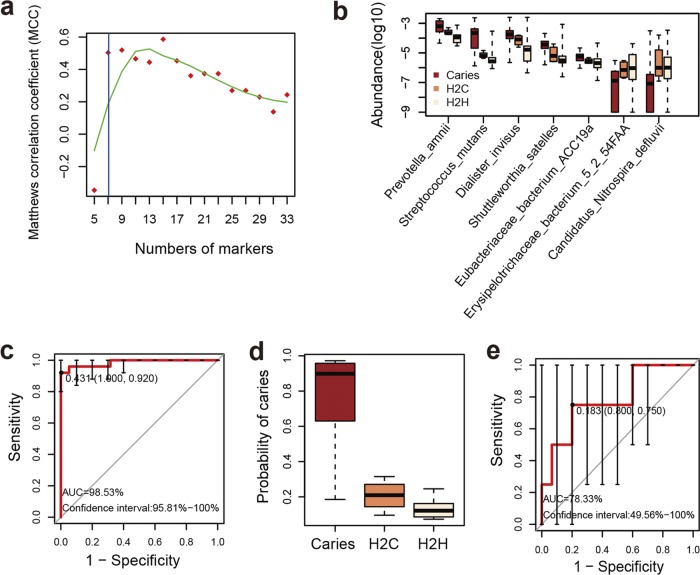
Classifier used to distinguish ECC children from healthy controls. (a) The mRMR method was used to identify the ECC-associated markers. Sequential subsets were generated at five-species intervals. For each subset, the error rate was estimated using a leave-one-out cross-validation of a linear discrimination classifier. Using only the seven marker species as predictors, the SVM model exhibited predictive performance that was already comparable to the performance of the model derived from the optimum (highest value of the Matthews correlation coefficient) subset. (b) The relative abundances of seven marker species among the H2H group, the H2C group, and the caries group are shown in a box plot. (c) Receiver operating characteristic (ROC) curves for the ECC group and healthy controls; 95% confidence intervals (CIs) are indicated by error bars. (d) The probability of caries determined by the classifier among the H2H group, H2C group, and caries group is shown in a box plot. (e) ROC curves for the H2H group and H2C group.

By use of the mRMR algorithm, 7 species markers were chosen to construct the SVM (support vector machine) classifier, which exhibited the best performance (Fig. 5a). Of them, 5 species (i.e., Streptococcus mutans, Prevotella amnii, *Eubacteriaceae bacterium* ACC19a, Shuttleworthia satelles, and Dialister invisus) were enriched in the caries group, as were “*Candidatus* Nitrospira defluvii” and *Erysipelotrichaceae bacterium* in the control group (Fig. 5b). This classifier manifested an area under the receiver operating characteristic curve (AUC) of 98.53% and a 95% confidence interval (CI) of 95.81% to 100% (Fig. 5c). Notably, the relative levels of the 7 marker species in the H2C subgroup all exhibited a tendency of approaching that of the caries group (Fig. 5d). We used the classifier to predict the future new ECC onset of these 19 healthy controls and showed that the classifier was able to predict the onset of caries with a moderately good performance (AUC = 78.33%) (Fig. 5e).

## DISCUSSION

Dental caries is a major oral health problem worldwide, affecting a great proportion of adults and children. The oral microbiome plays a crucial role in human health (19–22) and can profoundly affect the development of many diseases, including caries (20, 23). Microbial indicators of caries have been proposed as a method to predict future caries onset (24). There are approximately 700 prokaryotic species reportedly present in the human oral cavity, some of which may damage teeth under certain conditions (25). It has been reported that dental caries is directly caused by acid production on the enamel surface and that some microbes (e.g., Streptococcus mutans) play a significant role in this process. Dental caries is a polymicrobial disease that is not determined by one particular bacterium. Instead, it results from complex communal activities involving at least tens of bacterial species (26, 27). Thus far, however, a consensus has not been reached regarding cariogenic microbes and functional elements.

To study the effects of the oral microbiome on early childhood caries (ECC), we established an oral saliva gene catalogue (severe-ECC catalogue) from 44 children. The numbers of catalogue-specific genes were 22,824, 39,274, and 62,542 in the ECC group, healthy group, and HOMD-derived gene set, respectively. Our results revealed some differences between the oral microbiomes of the ECC and healthy groups. To facilitate subsequent analyses, we built an oral reference gene set by integrating our oral saliva genes with the gene set from the HOMD.

Our data revealed that the ECC and healthy groups exhibited considerable differences in taxonomic composition and functional profiles. For example, Prevotella amnii, Shuttleworthia satelles, Olsenella uli, and Anaeroglobus geminatus, whose connections with caries have not been reported, were enriched in the ECC group (Fig. 1e). The enrichment of Prevotella amnii in the ECC group is not surprising, as it is a species of the *Prevotella* genus, most of which have been reported to be related to caries and potentially have proteolytic/amino acid-degrading activities (12). Shuttleworthia satelles, Olsenella uli, and Anaeroglobus geminatus were reported to be present in the oral cavity and associated with periodontal disease (28, 29). Therefore, it appears that the presence of these microbes indicates an oral environment favoring caries onset. N. lactamica is considered a commensal microbe in the nasopharynx (30) and has been found to be the most abundant nasopharyngeal species in preschool children under the age of 5 years (31) (Fig. 1e). The higher levels of N. lactamica and Streptococcus australis in healthy subjects than in caries counterparts suggest that the two microbes correlate with dental health. Our results therefore not only corroborated previous findings on the relationship between oral microbiota and caries (14, 15, 17) but also identified new potential biomarkers of ECC.

Interestingly, StrainPhlAn analysis detected clear strain-level, but not species-level, differences in Actinomyces odontolyticus and Actinomyces graevenitzii between the caries and healthy participants (Fig. 2). Besides the taxonomic alterations, analysis of the cooccurrence network also indicated distinct patterns of interbacterial interactions in the saliva communities under healthy and ECC conditions (Fig. 3). For example, the main genera in the cooccurrence network were *Bacteroides* spp. and *Prevotella* spp. under the healthy condition, whereas *Streptococcus* spp. and *Prevotella* spp. were the main genera under the ECC condition.

Our analyses also revealed a prominent divergence between the caries and healthy groups at various functional levels. Functions related to carbohydrate metabolism were enriched in the ECC group, as revealed by the results of the gene profile, the KEGG orthology profile (Fig. 4a), and the eggNOG profile (Fig. 4b). This mirrored previous findings that enhanced carbohydrate activity in oral microbiota was a contributing factor of caries pathogenesis (32). In the membrane transport pathway, the phosphotransferase system was enriched in the ECC children (Fig. 4c), which indicated that transmembrane transport and phosphorylation were more active in this group. In the carbohydrate metabolism pathway, the citrate cycle (TCA cycle) was enriched in the healthy children, while glycolysis/gluconeogenesis was enriched in the ECC group. This result suggested that anaerobic metabolism of sugar was more active in the oral cavity of children with caries. At the module level, multiple metabolic components were enriched in the ECC group, including the AI-2 transport system, the PTS system, glucitol/sorbitol-specific II component, and nucleotide sugar biosynthesis (Table S5). Quorum sensing is an important mechanism underlying biofilm formation during the development of dental caries (32), which reportedly involves signal molecule autoinducer-2 (AI-2) in the interbacterial interaction (33, 34). While AI-2 is a quorum signaling component in some microbes, in other species the protein also harbors different activities (35) that may be implicated in caries development. The ABC transporter and two-component signal transduction system (TCS) regulates the expression of genes according to local environmental changes, which in turn influences bacterial competence, survival, and virulence. Moreover, the dramatic reduction of cell motility modules (Fig. 4b) in the ECC group could also be attributed to the increased biofilm formation in the ECC group, as inhibition of bacterial motility promotes biofilm formation (36).

Despite these findings, more research is needed to elucidate the precise mechanisms of oral microbiota in caries pathogenesis. Recent studies have shown that iron deficiency in young children is a risk factor for ECC (37), and this finding was confirmed in animal experiments (38). Our data revealed that iron complex transport was an enriched function in caries-free children, which was in agreement with previous results (Table S5). Other transport modules more enriched in the healthy group than in the caries group included sulfate transport, putrescine transport, and microcin C transport pathways (Table S5). It has been suggested that decreased activity of transporter proteins may lead to the accumulation of metabolic compounds, such as sugars and acids, and contribute to dental caries (37–39).

**FIG 6:**
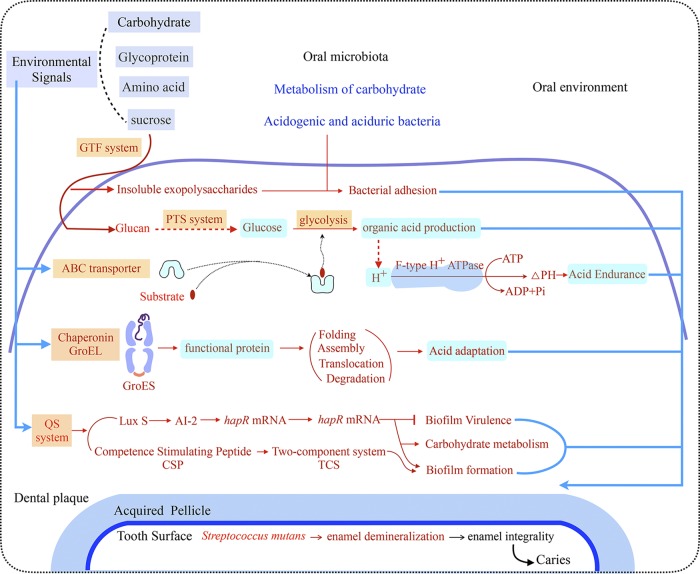
Taxonomic and functional characterization of oral microbiota in child caries. A schematic diagram shows the main functions of the oral microbes that are associated with caries. Red text denotes enriched functions in children with caries.

Our findings enabled us to propose a model to explain the roles of some microbes in caries pathogenesis (Fig. 6). It is known that dental plaque biofilms produce acids from carbohydrates that contribute to caries onset (40). Development of dental caries is a gradual process in which the first stage is characterized by oral biofilm formation and bacterial adhesion (41). Cariogenic bacteria, such as Streptococcus mutans, produce a GTF that synthesizes extracellular polysaccharides (EPSs) (42). The EPSs, especially water-insoluble glucans, play a critical role in dental plaque formation and biofilm stability, as these molecules allow cariogenic bacteria to adhere to enamel surfaces (43). The biofilm phenotype is regulated by its density-dependent quorum sensing (QS) system, which consists primarily of the competence stimulating peptide (CSP) and two-component signal transduction system (TCS) (44). In addition to biofilm formation, the CSP-mediated QS system also affects its acidogenicity and aciduricity (44). The PTS system is responsible for recognition, transmembrane transport, and phosphorylation of water-soluble glucans (45). When sugar is frequently consumed, glycolysis and acidification often ensue. F-type H+ ATPase and chaperonin GroEL may enhance the acidogenicity and acidurance of the cariogenic bacteria (46, 47). In the acidogenic stage, the acidogenic and aciduric bacteria rapidly propagate, whereby the demineralization/remineralization balance is tilted toward net mineral loss and leads to dental caries. In the aciduric stage, more aciduric bacteria, such as Streptococcus mutans and *Lactobacillus* spp., become dominant and aggravate the symptoms. As such, environmental acidification is a main contributor to caries development.

In summary, our results present differences in the oral microbial communities of healthy preschoolers and those with caries at various taxonomic and functional levels. As demonstrated by other microbiota association studies, understanding such complex and delicate relationships is crucial for the prevention and treatment of these diseases. Nevertheless, to better understand the microbial contribution in caries development, metatranscriptome analyses are needed and may provide additional evidence in elucidating the roles of taxonomic and functional variables in the oral microbiota.

Our conclusions from this study are as follows. (i) A microbiome gene catalogue from children with caries was constructed for the first time. (ii) Preschool children with dental caries and their healthy counterparts exhibited differences in oral microbiomes and functional profiles. (iii) The results demonstrate that multiple *Prevotella* spp. and *Veillonella* spp. are associated with dental caries and that the potential functional differences between children with caries and caries-free children are mainly distributed on carbohydrate metabolism functions/pathways. (iv) A panel of seven species was developed to predict the onset of caries.

## MATERIALS AND METHODS

### Study subjects.

Twenty-five children with severe ECC (decayed, missing, or filled tooth surfaces [dmfs] ≥ 8) and 19 caries-free (dmfs = 0) preschoolers, aged 45 to 73 months, were recruited in the study. Their diagnoses were made by a dentist at the Affiliated Hospital of Stomatology, Zhejiang University School of Medicine, according to the International Caries Detection and Assessment System II (ICDAS-II) (48). Written informed consent was obtained from the parents or other guardians of all participants prior to enrollment. The study was approved by the ethical committee of the Affiliated Hospital of Stomatology, Zhejiang University School of Medicine. We obtained consent to publish from the participant (or legal parent or other guardian for children) to report individual patient data, including images, videos, voice recordings, etc.

Exclusion criteria were as follows: (i) children with <18 teeth, (ii) children who received antibiotics or fluoride treatment in the prior 3 months, and (iii) children who suffered active bacterial or viral infections in other parts of the body (49).

### Saliva sampling and isolation of bacterial genomic DNA.

All subjects were asked not to eat or drink 2 h before sampling, which was performed in the morning. To minimize stimulation of salivation, saliva needed to be kept in the mouth for 3 min. Subjects were then instructed to drool into sterile cryogenic vials for 3 min. Each saliva sample was pipetted into a sterile 1.5-ml Eppendorf tube, which was snap-frozen in liquid nitrogen and stored at −80°C. Bacterial genomic DNA was extracted using the QIAamp DNA mini kit (Qiagen, Hilden, Germany) as previously described (10). To reduce contamination by human DNA, every 4 μg DNA was incubated with 160 μl MBD-Fc-bound beads from a NEBNext microbiome DNA enrichment kit (New England Biolabs, Inc., Ipswich, MA, USA). The enriched microbial DNA samples were purified by ethanol precipitation. DNA concentration and sizes were determined using NanoDrop and agarose gel electrophoresis. The resulting DNA samples were stored at –20°C until further processing.

### Illumina sequencing.

The metagenomic DNA libraries were constructed according to the Illumina TruSeq DNA sample prep v2 guide. The library insert sizes were checked using a DNA LabChip 1000 kit on a 2100 Bioanalyzer (Agilent Technologies, Santa Clara, CA, USA). All libraries were sequenced on a HiSeq 2000 instrument with the PE100 mode (Illumina, San Diego, CA, USA).

### Quality control of reads.

The following steps were used for quality control: (i) remove reads with more than 5 ambiguous bases and 50 low-quality bases (low quality indicated by a Phred quality score of less than 2), (ii) trim low-quality base tails of reads (low quality indicated by a Phred quality score of less than 2), and (iii) remove reads that were mapped to the human genome (HG19) by SOAPaligner 2.1 (50) using default parameters. The percentage of human reads accounted for, on average, 28% of the total sequencing data after the human DNA removal step. The microbial yield was apparently higher than the 20% to 30% proportion of microbial reads reported previously (19, 51).

### Genome assembly, gene prediction, and gene catalogue construction.

We carried out a two-round assembly strategy to improve the read utility ratios. For the first round, SOAPdenovo (52) (version 2.04) was used to assemble reads *de novo* for each sample, with parameters “−d 1 –M 3 –F” at k-mers ranging from 39 to 63, before the contigs with the longest *N*_50_ value were selected. For the second round, unused reads were selected by aligning clean reads with SOAPaligner 2.1 (50), prior to being repeatedly assembled with the same parameters at k-mer 59. MetaGeneMark (53) (prokaryotic GeneMark.hmm version 3.5) was used to predict open reading frames (ORFs) in contigs. The program predicted 3,086,934 ORFs with a length cutoff of 100 bp, and the total length of the ORFs was 1,548,170,042 bp. In light of the possibility that some low-abundance microbes were not detected in the limited sequencing data, we combined the previously public gene set from HOMD (http://www.homd.org/ftp/all_oral_genomes/20160329/) (54) to build a nonredundant oral gene catalogue for further analyses. This nonredundant gene catalogue was established by cd-hit-v4.6.1 (55) with parameters “−c 0.95 –aS 0.9 –r 0.” Redundant ORFs sharing 95% identity or greater and 90% coverage or greater were removed, resulting in a nonredundant gene catalogue composed of 2,200,443 genes.

### Profiling of microbial taxa and genes.

Organism and gene abundance were calculated according to previous studies (20, 56, 57). Briefly, clean reads were aligned against reference genomes and by SOAPalign2.21 with the parameters “−r 2 –m 100 –×1000.” Matched paired-end reads were chosen for further abundance calculation and then assigned to two types: (i) multiple reads that aligned to more than one species and (ii) unique reads that matched only one species. For species *S*, abundance Ab(*S*) could be divided into unique abundance Ab(*U*) and multiple abundance Ab(*M*). We then calculated Ab(*S*) as follows:

Ab(S)=Ab(U)+Ab(M)

Ab(U)=U/l

$$\mathrm{Ab}\left(M\right)=(\overset{M}{\underset{i=1}{\sum }}\text{Co}\times \left\{M\right\})/l$$

$$\text{Co}=\frac{\text{Ab}(U)}{\sum_{i=1}^{N}\text{Ab}(U)}$$

For each species (*S*), *U* and *M* are the number of unique and multiple reads, respectively, and *l* is the average genome length of species *S*. For each multiple read in {*M*}, there is a species-specific coefficient Co, and the *N* is the number of aligned species of this read.

Likewise, this method was also used to calculate gene abundance.

### MGS identification.

To cluster genes into metagenomic species (MGS), we applied the method described by Le Chatelier et al. (56), Qin et al. (20), and Nielsen et al. (57). First, gene markers with differential abundances were identified using wilcox.test in R (FDR < 0.07, Wilcoxon rank sum test corrected by the Benjamini and Hochberg method). Next, we clustered the marker genes using a Spearman’s correlation coefficient (rho) of >0.9 according to their abundances across all the individuals. After removal of clusters with fewer than 25 genes, a second hierarchical clustering was performed with the Spearman’s correlation coefficient between the mean abundance of genes in each cluster and a new threshold of 0.8. The final gene clusters were called MGS.

### Strain-level analysis.

StrainPhlAn was used for strain-level profiling. For each sample, clean reads were first mapped against the MetaPhlAn2 markers by Bowtie2 (58) to generate the consensus sequence, which represented the most abundant strain for each species in a sample. Similarly, the consensus sequences of public reference genomes of strains for each species were obtained by aligning the markers to these genomes. Finally, the extracted consensus sequences of references and samples were multiply aligned by MUSCLE (59), and the phylogenetic trees were built by RAxML (60) (parameters: −m GTRCAT and −p 1234).

### Gene function analysis.

Protein sequences were aligned to the KEGG gene database (KEGG release 71 July 2014) (61) and eggNOG v4.0 (62) by BLAT with parameters “−prot −out = blast8 −minIdentity = 30 −minScore = 60.” The best hit was selected for each gene based on score and identity. The abundances of eggNOG and KEGG orthologs were calculated as the sum of the abundances of all genes annotated to that ortholog.

Samples were functionally profiled using HUMAnN2 (http://huttenhower.sph.harvard.edu/humann2) (63). HUMAnN2 used the MetaCyc pathway database (https://metacyc.org/download.shtml) and MinPath to identify a parsimonious set of pathways which explain observed reactions in the community.

### Cooccurrence network.

To construct the metacommunity cooccurrence network, we first removed species with relative abundances of less than 0.01%. The Spearman correlation coefficients between species were computed using R, and all the *P* values were adjusted for multiple testing using the Benjamini and Hochberg false discovery rate (FDR)-controlling procedure. The cooccurrence networks were generated based on correlation coefficients (>0.6) and FDR (<0.05) for correlation and visualized by Cytoscape 3.0.2. Network properties were calculated with the igraph package.

### Association analysis between microbes and clinical variables.

To identify significant associations between oral microbial and phenotypic variables (see Table S1 in the supplemental material), we applied a statistical program of Multivariate Association with Linear Model (MaAsLin; https://huttenhower.sph.harvard.edu/maaslin) (64). In this study, age, gender, and BMI were included as potential confounders in each model. To test the association for each species, we first filtered low-abundance species and confined our analysis to 205 species that had relative abundances of >0.01% and were present in more than 10 individuals. These 205 species accounted for, on average, 99.3% of microbial reads. The percentage of each species was arscine-square-root transformed by taking the arcsine of the square root of the proportional value of each species. For MetaCyc pathways, the same filtering criteria were used, and a total of 315 pathways were further associated with different factors using MaAsLin. In each analysis, the false discovery rate was controlled at a *q* value of 0.1 using the Benjamini and Hochberg method (p.adjust package in R).

### Classifier construction.

We used an SVM (support vector machine) (R 3.1.3; the e1071 R package) to build the classifier for ECC. The differentially abundant species (*P* < 0.01) were chosen as features. To filter out redundant features, the mRMR algorithm (65) (the sideChannelAttack R package) and the leave-one-out cross-validation LDA (linear discriminant analysis) (the paleoMAS R package) were applied. The feature set which has the highest Matthews correlation coefficient (MCC) was chosen to build the SVM classifier. The receiver operating characteristic (ROC) figures were drawn by using the pROC R package.

### Statistical analyses.

To detect significant differences in relative abundance of metagenomics features, the nonparametric Wilcoxon test (wilcox.test package in R) was performed. The FDR was calculated using the Benjamini and Hochberg method (p.adjust package in R).

### Data availability.

The Illumina raw read data have been deposited at the National Center for Biotechnology Information (NCBI) under accession number SRP103050.
