# Supplementary figures and images for: Oral Microbiome Alterations Associated with Early Childhood Caries Highlight the Importance of Carbohydrate Metabolic Activities
Source: mSystems. 2019 Nov 5;4(6):e00450-19. doi: 10.1128/mSystems.00450-19 (PMC6832018; doi:10.1128/mSystems.00450-19)

**a**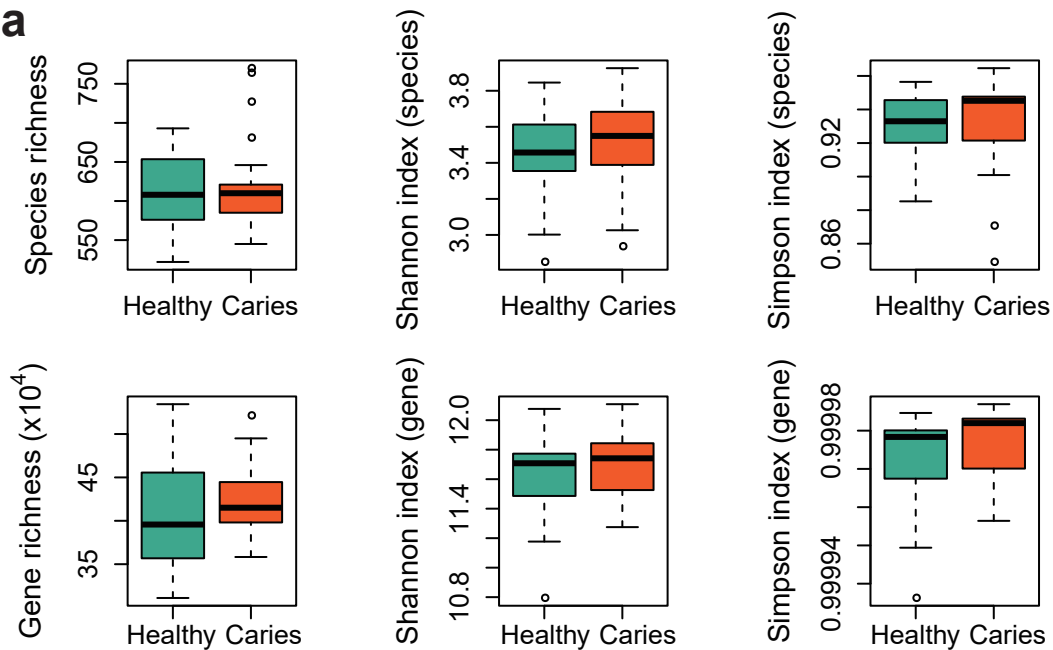**b**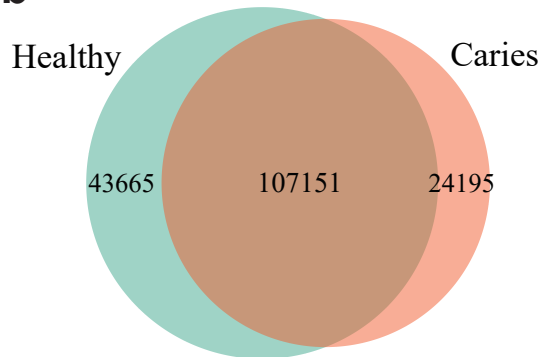**c**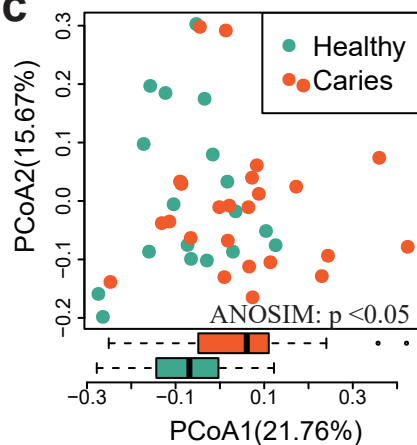

Supplement: FIG S1 [file mSystems.00450-19-sf001.pdf]

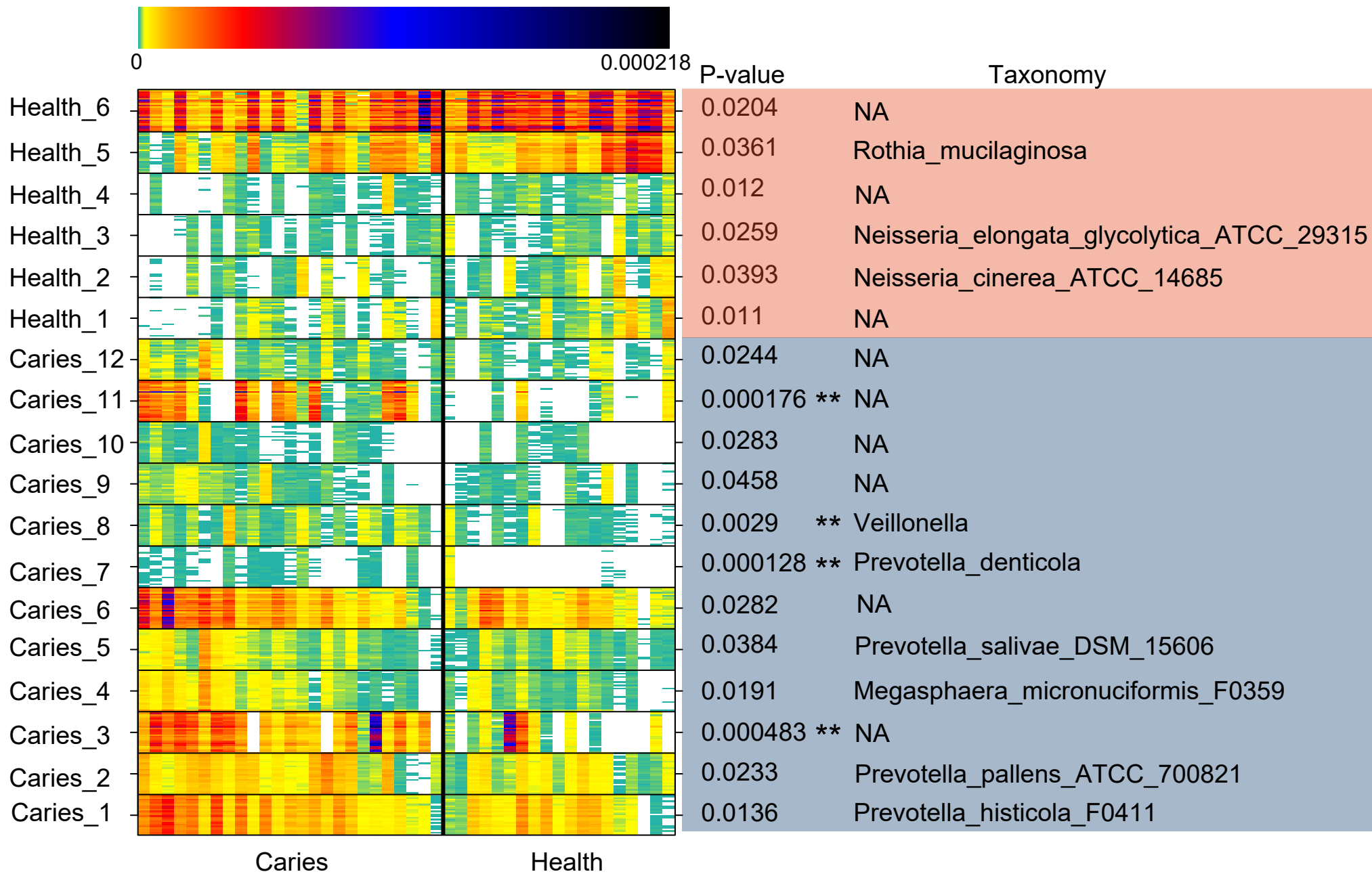

Supplement: FIG S2 [file mSystems.00450-19-sf002.pdf]
